# Triggers, Types, and Treatments for Kounis Syndrome: A Systematic Review

**DOI:** 10.3390/clinpract15030059

**Published:** 2025-03-13

**Authors:** Erick Rochel-Perez, Miguel Santaularia-Tomas, Mario Martin-Dorantes, Edgar Villareal-Jimenez, Amonario Olivera-Mar, Ely Sanchez-Felix, Adrian Perez-Navarrete, Jose Luis Millet-Herrera, Osvaldo Huchim-Mendez, Ricardo Alejos-Briceño, Nina Mendez-Dominguez

**Affiliations:** 1 School of Medicine, Universidad Marista de Merida, Merida 97300, Mexico; erochel2114102@a.marista.edu.mx (E.R.-P.); aperez1914093@a.marista.edu.mx (A.P.-N.); jmillet1914078@a.marista.edu.mx (J.L.M.-H.); ralejos2214025@a.marista.edu.mx (R.A.-B.); 2 Hospital Regional de Alta Especialidad de la Peninsula de Yucatán, IMSS-BIENESTAR, Merida 97130, Mexicoamar.hraepy@imssbienestar.gob.mx (A.O.-M.); elysanchezfelix@gmail.com (E.S.-F.); 3 Health Sciences School, Universidad Autonoma de Yucatán, Merida 97203, Mexico; a23220303@alumnos.uady.mx; 4 School of Medicine, Universidad Anahuac, Mexico City 97310, Mexico

**Keywords:** Kounis syndrome, allergic myocardial infarction, treatment outcome, precipitating factors, systematic review

## Abstract

**Background**: Kounis syndrome (KS), also known as allergic myocardial infarction, presents in three variants. This condition is often underrecognized due to limited knowledge and its variable presentation. To address these limitations, the present review aims to describe the triggers, types, management, and patient outcomes of KS. **Methods:** In this systematic review, PubMed and Scopus were used to identify publications of clinical case reports; variables included sociodemographic characteristics, clinical manifestations, triggers, treatments, and outcomes. Data from the articles´ abstracts were assessed by two corresponding authors, and subsequently, each case was analyzed by two coauthors, validated and analyzed with Stata 12. To categorize each Kounis type, mean and proportion comparison tests were performed, and measures of association were obtained using logistic regression and expressed as odds ratios. **Results:** A global distribution was identified, with predominance in the Northern Hemisphere. Type I KS was the most reported variant, and most of the patients were adult men. Most of the patients presented variability in the treatment and outcomes. **Conclusions:** KS may represent a diagnostic challenge, and underdiagnosis could explain the lack of uniformity in the diagnostic and assessment process. Our results highlight a need for improved approaches based on patient history for correct diagnosis and preventing recurring events.

## 1. Introduction

Kounis syndrome (KS) was first described by Kounis and Zavras in 1991 [1]. This disease, also known as allergic myocardial infarction or allergic angina syndrome, is defined as the presence of an acute coronary syndrome with the concomitant activation of mast cells and platelets in the context of a hypersensitivity reaction, triggered by an allergic, anaphylactic, or anaphylactoid event [2,3]. The increase in inflammatory mediators—such as histamine, products derived from arachidonic acid, platelet-activating factors, neutral proteases, and different cytokines and chemokines—can cause coronary vasospasm, erosion, or rupture of an atheromatous plaque or coronary thrombosis, leading to myocardial infarction [3,4].

KS occurs as an acute response when an allergic reaction is associated with acute coronary syndrome; its pathophysiology is explained as a response involving different cells that cause anaphylaxis. Mast cells and basophils play a fundamental role in IgE- and non-IgE-mediated mechanisms. IgG and MRGPRX2 may also intervene according to recent findings. Mediators in the cardiovascular system generate coronary vasoconstriction mediated by histamine, and pre-existing atheromatous plaques may translate into pain and initial clinical manifestations. Lipid mediators cause vasoconstriction associated with angina and platelet-activating factor and lead to circulatory failure [2,3].

Three variants of KS have been described. They share the same initial pathophysiological mechanism and results but differ in terms of coronary damage involved and the presence or absence of comorbidities in the patient. The type I variant is the most common, characterized by coronary obstruction secondary to vasospasm, in patients without coronary artery disease or predisposing factors. The type II variant refers to KS in patients with pre-existing atheromatous coronary artery disease in whom the inflammatory reaction may induce coronary vasospasm or rupture of the atheromatous plaque, consequently causing myocardial infarction. Finally, the type III variant occurs in patients with coronary stents, where the activation of inflammatory mediators leads to stent thrombosis, which can cause acute myocardial infarction [2,5,6].

KS is not necessarily a rare condition; rather, it is often underrecognized due to a lack of awareness of its diagnostic criteria and the variability in its clinical presentation. KS can be easily misdiagnosed, and therefore, the number of reported cases of this disease is underestimated. Failure to establish a diagnosis of KS has reduced the amount of information available about this syndrome, including its treatment options and their effectiveness [2,7]. The objective of the present review is to provide an aid for clinicians to fill in the gaps in the understanding of KS by (a) describing the epidemiology of the three variants of KS and their triggers; (b) analyzing the influence of different sociodemographic factors on the presentation of the pathology; (c) clarifying the diagnostic criteria and describing the most common electrocardiographic findings; and (d) standardizing the therapeutic options available for the treatment of KS through the analysis of the results of different medical approaches.

## 2. Materials and Methods

### 2.1. Search Strategy

The present study is a systematic review with meta-analysis for which the criteria established in the PRISMA guidelines were followed [8] (please see Supplementary Table S1 for PRISMA checklist). PubMed and ScienceDirect were used to identify the information, and included articles published between 2018 and 2023 were selected, which were case reports or case series that met the inclusion and exclusion criteria of single case reports in humans. To conduct the search, the term “Kounis syndrome” was used, which had to be present in the title or in the abstract of the article. The protocol guiding the present review was registered in the PROSPERO International Prospective Register of Systematic Reviews (CRD 42024499452); a direct link for the results of the search can be found at https://pubmed.ncbi.nlm.nih.gov/rss/search/1HIKpXrSMrog0zZIIx-w21UUsMwElJKcHUauqhtKP3KUQCJ95w/?limit=15&utm_campaign=pubmed-2&fc=20231220140553 (accessed on 12 January 2025). For a representative case inclusion, we developed a search using Scopus and added all publications meeting the inclusion criteria that were not already added from PubMed.

### 2.2. Selection Criteria

**Figure 1:**
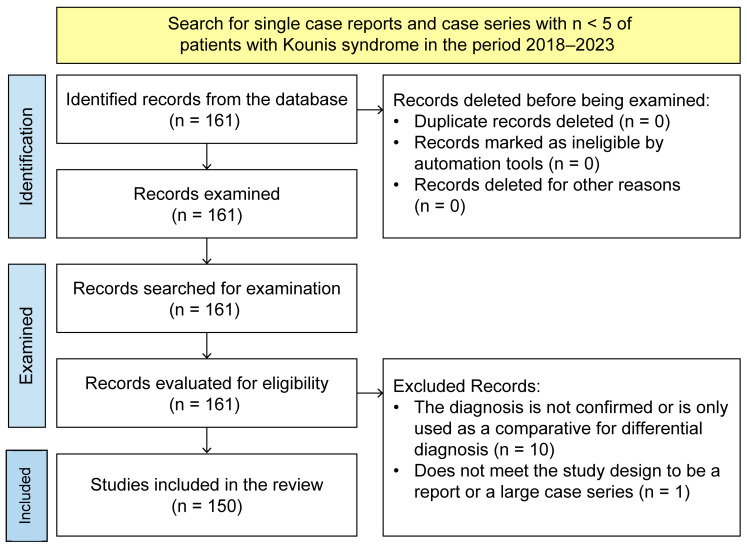
Study selection flowchart for Kounis syndrome (KS) case reports published in PubMed and Scopus.

Reports or case series reporting < 5 patients were to be included if the presence of KS was identified as a diagnosis of acute coronary syndrome present in patients; publications in English and Spanish indicating KS as the final diagnosis were included. The initial search resulted in 161 articles, of which, after automatically excluding repeated publications using Excel, the corresponding authors independently reviewed the pertinence of each article, registered comments in the Excel dataset, and indicated which publications were unrelated to the topic. In the second phase, a total of 150 articles were reviewed by coauthors by selecting a proportional number of publications to be thoroughly analyzed, as shown in Figure 1. Each case report was reviewed by two coauthors, and their annotations were recorded in the Excel spreadsheet. In the third phase, the coauthors’ annotations were revised and validated using ES-F and EVJ.

### 2.3. Data and Variable Extraction

The pertinent data of the articles were collected with the Microsoft Excel^®^ program using a collection format that contains the selected articles and variables. From the case reports, authors’ data, date of publication, sociodemographic variables, and clinical variables were extracted. Sociodemographic variables include patient age, sex, and geographical region of origin.

### 2.4. Clinical Variables

- KS classification: The classification assigned to patients based on the three forms of presentation of the syndrome.
- The causative agent: The etiological agent that causes allergic reactions.
- Therapeutic approach: The treatment they received during the hospital stay.
- Days of hospital stay.
- Electrocardiogram findings: ST-segment elevation, ST-segment depression, T-wave inversion, normal, and other findings (sinus tachycardia, ventricular fibrillation, prolonged QT segment, sinus bradycardia, AV block, biphasic T-waves, etc.).
- Laboratory results: These are used to identify cardiac (cardiac enzymes) and immunological involvement (serum tryptase levels, IgE, histamine, or mast cell degranulation).
- Imaging tests: These are performed if angiography and/or a CT scan was performed.
- The presence of acute myocardial infarction: Necrosis of the myocardium caused by an obstruction of the blood supplied to the heart [9].
- The outcome of the emergency.

### 2.5. Statistical Analysis

All data were synthesized and recorded in a Microsoft Excel dataset and exported to Stata 12 for further analysis. Descriptive graphs and tables were generated for the total sample and divided according to age group, classification of KS, triggering factor, patient evolution, treatment, and result [10].

Comparisons between groups were performed using mean comparison tests and chi-squared tests for contrasting the frequency or mean, respectively, of a variable against the other types of KS. Significant differences are presented only in text. Measures of association were obtained using logistic regression for binary dependent variables, with the three KS types as dependent variables, and sociodemographic characteristics, approach, triggers, and outcomes as independent variables. We also performed a multinominal regression to associate odds ratios (ORs) for each Kounis type with the other two types and used an unspecified type as a reference. In all cases, *p* < 0.05 was established as statistical significance. Risk of bias due to missing evidence in a meta-analysis was assessed using the ROBINS-E tool [11].

## 3. Results

**Figure 2:**
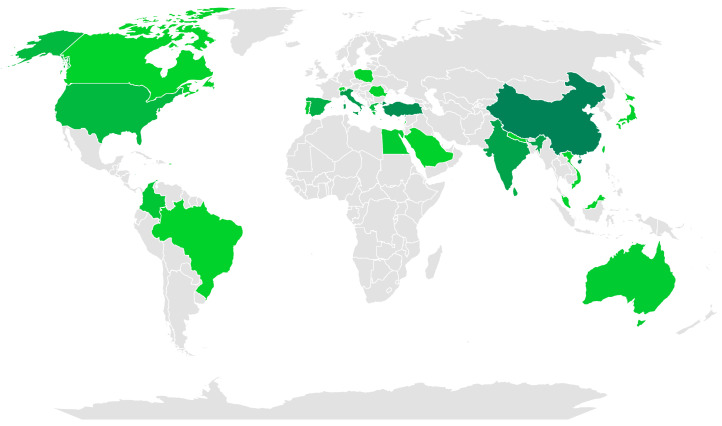
World distribution of case reports regarding Kounis syndrome. The number of published case reports is presented in green shades. Darker shades indicate more published cases reported.

A total of 150 publications, incorporating 155 cases of patients, were included between reporting and single cases or less than three cases, from 2018 to 2023, of which 21.47% belong to the year 2023. A global distribution was found, with the greatest predominance in the Northern Hemisphere, and with the United States being the country with the highest number of cases (27/155), followed by Japan (25/155), Italy (17/155), China (15/155), and Turkey (14/155), as shown in Figure 2.

For the 150 published articles, the mean age was 54.3 years; KS was presented predominately in men; and in 67.5% of the reported cases, 26.38% were type II and 14.11% type III. However, 13.49% of the cases did not specify any type. Of all the reports, survival was reported for 99 patients, of whom 92 (57.5%) presented acute myocardial infarction but survived (AMI), and 7 died.

### 3.1. Type I

In patients with type I KS, the mean age was 49.18 years (*p* = 0.009), and 57.33% were men (*p* = 0.01); thus, compared to patients with types II and III, patients with type I were significantly younger and included a higher proportion of women. The most frequent triggering factor was drugs (49.33%), which included antibiotics, benzodiazepines, and serotonin receptor (5-HT₃) antagonists. In electrocardiographic findings, ST-segment elevation was present in 66.66% of cases, followed by ST-segment depression in 44.66% of cases (*p* = 0.038). Among the most used treatments were anti-allergy treatments (72%), which were more frequently used in patients with types II and II but equal to the percentage of anti-allergy treatments registered for patients with an unspecified Kounis type. Corticosteroids were used in 62.5% of patients, and non-pharmacological treatments were used in 37.30%. The outcome of death of the patient was reported in 1 out of 71 patients with type I, which represents 1.4%.

### 3.2. Type II

In patients with type II KS, the mean age was 62.69 years (*p* = 0.002), and 86.04% were men (*p* = 0.022). Compared to patients with type I, patients with type II were significantly older and included a higher proportion of men. The most common causes were drugs (37.02%) and insect and animal venom (37.02%); there were significantly fewer cases triggered by drugs in type II compared to types I and II. ST-segment elevation occurred in 63.79% of cases, followed by ST-segment depression in 27.90% of cases. Regarding treatment, a preference for cardiovascular management was observed (66.66%), followed by non-pharmacological interventions (65.11%, *p* = 0.009) and the use of anti-allergy drugs (57.77%). There were no deaths reported among these patients.

### 3.3. Type III

In patients with type III KS, the mean age was 61.3 years, and 78.26% were male compared to patients with type I; patients with type III were significantly older and included a higher proportion of men. The most frequent triggers were drugs (43.47%), contrast medium (13.04%), and objects (13.04%). In the electrocardiogram, ST-segment elevation was the most common abnormality, observed in 82.60% of cases, which is significantly higher than the percentage observed in cases of types I and II. ST-segment depression occurred in 8.69% of cases (*p* = 0.004), which is a significantly lower proportion than that observed for types I and II. Non-pharmacological management was the first choice (65.21%), followed by anti-allergy management (60.89%) and cardiovascular management (60.89%). Death occurred in 4 of the 22 patients reported as having type III (*p* = 0.009), which corresponds to 18%, meaning that patients with type II Kounis had fatal outcomes more frequently than those with types I and II and those with unspecified types.

In case reports without a reported type of KS, the mean age was 49.95 years, and 59.09% were men. The most frequent causes were drugs (40.90%) and insect and animal venom (36.36%). The most frequent electrocardiographic abnormality was ST-segment elevation, observed in 59.09% of cases, followed by ST-segment depression in 40.90% of cases. Treatment was mainly pharmacological with anti-allergy drugs (72.72%), followed by corticosteroids (68.18%) and cardiovascular management (59.09%). The non-pharmacological approach was used in 40.09% of cases, which is significantly lower than the percentage of non-pharmacological treatment used for types II and III, but no significant difference was found when compared to type I. Non-pharmacological treatment was applied in combination with another pharmacological treatment. The mortality outcome was observed in 2 of the 22 cases, representing 9%.

Among the triggering factors, prescription drugs were the most frequent, followed by insect or animal venom or contrast medium, as shown in Table 1. Among the tests requested at the time of care, it was identified that 97.74% had an electrocardiogram, and 84.37% had laboratory test results. Moreover, 94.1% of the patients had more than one electrocardiographic abnormality, with the most frequent being ST-segment elevation in 64.51% (n = 100), ST-segment depression in 35.48% (n = 55), and T-wave inversion in 11.61% (n = 18) of the patients.

The most frequently requested laboratory test was the detection of cardiac enzymes in 87.7% of cases, such as troponins and creatine phosphokinase (CPK), and 29.03% of patients underwent immunological response tests, which were tryptase levels, immunoglobulin E (IgE), histamine, and mast cell degranulation tests. The most frequently used imaging studies were angiography (n = 119, 76.77%) and computed tomography (n = 19, 12.25%). The most used drugs were anti-allergy drugs, including H1 and H2 antihistamines, corticosteroids (69.03%), and cardiovascular drugs (60%), such as nitrates, antiarrhythmics, antiplatelet agents, anticoagulants, and thrombolytics. Non-pharmacological treatments included the use of procedures such as catheterization, life support, oxygen therapy, and hydro-electrolyte management.

Table 2 shows the multinominal regression model, including the factors associated with the univariate regression analysis, with sex and age being the characteristics associated in the three types, having a greater relationship with type II, with an OR of 3.99; in relation to the outcome in type III, an OR of 9.40 was found, while independent associations for each Kounis type are presented in Odds Ratio in Table 3, where a significant association was found between allergic reaction to objects and KS type III. 

Finally, by using a risk of bias assessment for observational studies, the risk of bias for the present non-randomized study was categorized as low. The bias relied mainly on missing unspecified characteristics of patients and classifications of KS type in single case reports; bias due to selection randomization or unreported statistical data does not apply as we included all published case reports.

## 4. Discussion

KS is characterized by an inflammatory response derived from the activation of mast cells and platelets, which results in acute coronary syndrome. Although its pathophysiology is understood, the heterogeneity of the triggers can make its clinical diagnosis difficult. In addition, there is no established consensus to systematize diagnostic and therapeutic approaches for these patients [4,11,12]. Similarly to the findings in the present review, Cahuapaza-Gutierrez and colleagues identified a mean age of 54.4 years and predominance in men [13].

In the cases analyzed, a higher frequency of type I KS was identified, followed by type II and type III, with a mean age of 54 years and a predominance in males. These findings are consistent with the existing literature, which documents a higher incidence of type I KS in men of around this age [3,14]. However, we would like to emphasize that the same patient may present with more than one type of KS, and although this presentation is infrequent, its recognition is crucial since it could optimize clinical decision making and contribute to improving the prognosis through personalized and precise strategies.

Type I KS is characterized by the presence of coronary spasms in individuals with morphologically normal coronary arteries and no risk factors for coronary heart disease. In contrast, type II occurs in patients with a history of atherosclerotic disease. This distinction suggests that an epidemiological characterization between both subgroups would improve the understanding of the prevalence and distribution of KS. Considering that coronary heart disease is a leading cause of morbidity and mortality worldwide, implementing specific identification strategies for each type is essential, even in developed countries such as the United States, where its prevalence is higher in men and increases with advanced age [15]. In developing countries, reported cases of KS are limited; however, the proportion of the different subtypes of KS is likely to differ from that documented in the literature due to the variability in the predominant risk factors in these regions.

The burden of coronary heart disease in America becomes evident when considering data reported by the Pan American Health Organization (PAHO), which indicate that in this region, in 2019, ischemic heart disease and diabetes mellitus were the two main causes of mortality in the total population [16]. In the study conducted by López-Jaramillo and López-López on the causes of mortality in urban and rural populations of Argentina, Brazil, Chile, and Colombia, the main cause of death was cardiovascular disease; diabetes mellitus, arterial hypertension, and smoking were among the factors identified in their study population that contributed to it [17]. These data make us think more about the high possibility of variability in the proportion of KS variants in developing countries, with type II probably being the most common.

Cardiovascular disease is the main cause of death internationally, and low-income countries such as Venezuela, Colombia, Ecuador, Brazil, and Mexico have a greater number of deaths related to cardiovascular disease. The main risk factors for ischemic heart disease and coronary vascular damage include arterial hypertension, diabetes mellitus, smoking, and dyslipidemia, which are also linked to the typical profile of patients with type II KS [18,19,20].

As reported in previous studies, the cases included in this study were predominantly registered in developed countries such as the United States, Japan, Turkey, China, and Italy, while incidences were notably lower in developing countries such as Mexico and Brazil. This disparity could be attributed to the lack of knowledge and recognition of KS in these regions, limiting its timely diagnosis and adequate management. Therefore, it is imperative to promote medical education for general practitioners, emergency physicians, and cardiologists to improve early identification of the syndrome, which would allow for more effective therapeutic intervention and potentially better clinical outcomes for affected patients [4].

As described in the literature, there are multiple causes; however, it was identified that the main trigger is due to medications, although other triggers such as insect bites, food intake, and vaccination have also been recognized, especially the SARS-CoV-2 vaccine. KS associated with this vaccine has been reported in previous studies, especially in elderly patients with comorbidities, which make them more susceptible to developing coronary artery disease and therefore having increased susceptibility to this syndrome [3,21,22]. Another scenario for risk was presented in a systematic review by Dai and colleagues, who explained that coronary vasospasm is often less frequently recognized as a form of allergic manifestation in the perioperative setting [3,23]. 

The diagnosis of KS is made by a strong clinical suspicion of a previous anaphylactic reaction in the context of acute coronary syndrome. Thus, an important element for its correct diagnosis is a complete clinical history, inquiring about allergen exposure and history of clinical symptoms of anaphylactic reaction. The electrocardiographic findings in the present study include changes in the electrocardiogram suggestive of ischemia; the most frequent was ST-segment elevation [14]. In a systematic review with a case report, Paratz and colleagues reported a mortality of 22%, which is higher than what we observed from reviewed patient cases; the authors explained that cardiac manifestations were predominantly reported in male patients, which coincides with our present findings. Additionally, they presented repeated cardiac arrests in a 72-year-old patient and proposed management of patients with severe mastocytosis. Their guidelines could be useful for the future integration of fluxograms and protocols [24].

Laboratory study results are useful for the diagnosis of KS because the concomitant presence of cardiac enzymes and elevated immunological molecules helps to link the presence of cardiovascular abnormality to an immunological one. In most cases, elevated levels of troponins and CPK were reported; however, tryptase and other markers of anaphylactic response were used in smaller proportions [4]. The tryptase released by mast cell degranulation is involved in anaphylactic reactions, and high concentrations of this enzyme can be associated with anaphylactic shock and death; therefore, its measurement in the context of KS could help to assess the severity of patients, although the short half-life of KS should be considered [3,25].

For the analysis of coronary anatomy in the patients with KS included in this study, most underwent coronary angiography, and only a small percentage underwent computed tomography angiography. Although both imaging studies provide reliable information on coronary anomalies, considering the initial use of computed tomography angiography could benefit patients by avoiding invasive studies that provide the same anatomical information about the coronary arteries [26]. However, these findings may differ from those reported in the systematic review by Giovanni et al. including pediatric patients, in which new definition criteria were proposed considering the presence of allergic myocardial infarction by considering this manifestation as relevant for diagnosis in children [27].

A need for awareness of KS is also manifested in certain reviewed therapeutic approaches. Multivariability was identified in the drugs used for the treatment of patients despite the recommendation of the use of H1 and H2 antihistamines and corticosteroids in type I and type II KS for the control of anaphylactic response; these were used in only 66% and 60% of the cases, respectively. Calcium channel blockers are especially useful for resolving coronary vasospasm caused by hypersensitivity reaction but were only used in 12% of cases [3,4,13,28].

The limitations of the present study are the retrospective nature of the design and the fact that only studies where the diagnosis was identified were included; ideally, prospective studies could include a comparison group. KS has no ICD-10 coding; therefore, incidence based on epidemiological data of hospitalizations and deaths could not be included in the Introduction as part of the rationale.

## 5. Conclusions

KS may often be underrecognized due to a lack of awareness of its diagnostic criteria and variability in its clinical presentation. Patterns of reported clinical cases reveal regions where the syndrome is less diagnosed, with potential difficulties in correct classification and treatment. To increase the timely detection of KS, it remains important to raise awareness of its diagnostic criteria. Through correct diagnoses and the dissemination of case reports, in the near future, the true prevalence may be estimated, and therefore, protocols could be improved for the treatment of patients with this syndrome. There is a need for prospective and multicenter studies from every geographical region to ultimately develop guides and recommendations that consider the variability of human groups.

## Figures and Tables

**Table 1:** Sociodemographic data and clinical variables of 155 patients with Kounis syndrome reported in 150 case reports.

Variable	Frequency	Mean/Percentage
Sociodemographic Data		
Age (years)	150	54.37
Not reported	5	7.8%
Male	100	67.50%
Female	50	32.50%
Not reported	5	7.8%
Type		
Type I	71	46.01%
Type II	41	26.38%
Type III	22	14.11%
Not reported	21	13.49%
Trigger		
Prescription drug	71	44.37%
Poison	44	27.50%
Contrast medium	16	10.00%
Food	11	6.87%
Vaccine	9	5.62%
Object	4	2.50%
Unspecified	4	2.50%
Other	2	1.25%
Tests Performed		
Electrocardiogram	151	97.41%
Cardiac enzymes	136	87.74%
Tryptase immune response tests	45	29.03%
Angiography	119	76.77%
Tomography	19	12.25%
Outcomes		
Death	7	4.37%
Myocardial infarction	92	57.50%
Approach and Treatment		
Allergic management	107	69.03%
Antihypertensive management	25	12.62%
Lipid-lowering management	24	15.00%
Cardiovascular management	96	60.00%
Corticosteroids	97	60.62%
Antibiotic	7	4.37%
Antimuscarinic	7	5.00%
Non-pharmacological treatment	77	48.12%
Other	15	9.37%

**Table 2:** Multinominal logistic regression indicating variable association between type of Kounis syndrome and related triggers. Each type is presented as dependent variable and is compared with other types, and unspecified type is used as reference.

Variable	Odds Ratio (OR)	Standard Error	p	95% Confidence Interval	
Type I					
Sex (male)	**0.41**	0.14	**0.011**	0.20	0.81
Age	**0.96**	0.00	**0.001**	0.94	0.98
Drug	1.61	0.51	0.137	0.85	3.02
Contrast medium	0.48	0.27	0.194	0.15	1.45
Vaccine	0.54	0.39	0.408	0.13	2.27
Food	2.08	1.35	0.257	0.58	7.42
Poison	0.71	0.25	0.353	0.35	1.44
Object	0.36	0.43	0.393	0.03	3.62
Death	0.17	0.19	0.114	0.02	1.51
Type II					
Sex (male)	**3.99**	1.91	**0.004**	1.56	10.21
Age	**1.04**	0.01	**0.001**	1.01	1.07
Drug	0.71	0.26	0.361	0.34	1.46
Contrast medium	1.73	0.95	0.317	0.58	5.10
Vaccine	0.76	0.63	0.746	0.15	3.84
Food	0.25	0.27	0.199	0.03	2.05
Poison	1.88	0.72	0.098	0.88	3.98
Infarct	1.03	0.37	0.921	0.51	2.10
Type III					
Sex (male)	1.88	1.00	0.239	0.65	5.38
Age	**1.03**	0.01	**0.043**	1.00	1.06
Drug	1.01	0.46	0.971	0.41	2.47
Contrast medium	1.43	0.97	0.601	0.37	5.47
Vaccine	1.76	1.47	0.495	0.34	9.09
Food	1.35	1.10	0.71	0.27	6.70
Poison	**0.21**	0.16	**0.044**	0.04	0.96
Object	**20.4**	24.05	**0.011**	2.02	205.79
Infarct	1.83	0.88	0.211	0.70	4.74
Death	**9.40**	7.54	**0.005**	1.95	45.29

**Table 3:** An independent logistic regression analysis of the three variants of Kounis syndrome. Each type is independently associated with the other two types in separate models.

Variable	Odds Ratio (OR)	Standard Error	*p*	95% Confidence Interval	
				Lower	Upper
Type I					
(Log likelihood: −93.80; pseudo R^2^: 0.13)					
Sex (male)	**0.33**	0.12	**0.005**	0.15	0.71
Age	**0.96**	0.01	**0.001**	0.94	0.98
Drug	1.89	0.72	0.096	0.89	4.02
Contrast medium	0.73	0.50	0.656	0.19	2.82
Food	3.15	2.28	0.114	0.75	13.07
Death	0.21	0.24	0.173	0.02	1.96
Type II					
(Log likelihood: −73.84; pseudo R^2^: 0.19)					
Sex (male)	**5.97**	3.26	0.001	2.04	17.41
Age	**1.05**	0.01	**0.000**	1.02	1.08
Food	0.23	0.25	0.19	0.02	2.05
Poison	1.96	0.86	0.129	0.82	4.67
Type III					
(Log likelihood: −52.87; pseudo R^2^: 0.19)					
Sex (male)	2.57	1.62	0.132	0.75	8.85
Age	**1.03**	0.01	**0.043**	1.00	1.06
Poison	0.23	0.18	0.066	0.05	1.10
Object	**24.86**	32.77	**0.015**	1.87	39.19
Death	6.91	6.96	0.055	0.95	49.83

## Data Availability

The database is included in the Supplementary Materials.

## Supplementary Materials

The following supporting information can be downloaded at https://www.mdpi.com/article/10.3390/clinpract15030059/s1, Table S1: Final search of included case report publications and references [29].
